# Racial and ethnic disparities in polycystic ovary syndrome

**DOI:** 10.1016/j.fertnstert.2023.01.031

**Published:** 2023-01-23

**Authors:** Katherine VanHise, Erica T. Wang, Keith Norris, Ricardo Azziz, Margareta D. Pisarska, Jessica L. Chan

**Affiliations:** a Division of Reproductive Endocrinology and Infertility, Department of Obstetrics and Gynecology, Cedars-Sinai Medical Center, Los Angeles, California; b Division of General Internal Medicine and Health Services Research, University of California, Los Angeles Medical Center, Los Angeles, California; c Department of Obstetrics and Gynecology, Heersink School of Medicine, University of Alabama at Birmingham, Birmingham, Alabama; d Department of Medicine, Heersink School of Medicine, University of Alabama at Birmingham, Birmingham, Alabama; e Department of Health Policy, Management and Behavior, School of Public Health, University at Albany, SUNY, Rensselaer, New York; f Department of Healthcare Organization and Policy, School of Public Health, University of Alabama at Birmingham, Birmingham, Alabama.

**Keywords:** PCOS, race, ethnicity, disparities, polycystic ovary syndrome

## Abstract

Polycystic ovary syndrome (PCOS) is a common endocrine disorder that impacts women worldwide. There are several racial and ethnic differences in PCOS phenotypes and in PCOS- associated metabolic dysfunction. In this review, we summarize the current literature on disparities in the diagnosis and outcomes associated with PCOS in the United States. Future studies are needed to address gaps in knowledge for racial and ethnic-specific differences in PCOS, and include a large number of non-White and/or Hispanic participants in PCOS studies.

Polycystic ovary syndrome (PCOS) is a highly prevalent endocrine disorder that impacts reproductive-age women worldwide. The defining features of this disorder include irregular menstrual cycles, clinical and/or biochemical hyperandrogenism, and the finding of polycystic ovarian morphology on ultrasound examination. The most current 2018 international guidelines require the presence of ≥2 of the 3 aforementioned criteria to establish a diagnosis of PCOS, after exclusion of related endocrine disorders (1). A genetic basis for PCOS is suggested by a strong resemblance seen among twins, with one Dutch study finding a 71% correlation of PCOS in monozygotic twins and 38% correlation of dizygotic twins (2). Genome wide association studies have identified 19 specific genetic susceptibility loci in women with PCOS (3). Eleven of these loci were identified in women of Han Chinese and European descent, suggesting that even between different races there may be a common genetic basis for the disease (3–5). However, the loci identified from genome wide association studies can only account for <10% of PCOS hereditability, thereby implying that there are likely epigenetic, socioeconomic, cultural, and/or environmental factors that lead to the development of PCOS (3).

Several studies have demonstrated global differences in PCOS phenotypes of women of different racial and ethnic groups. For example, Middle Eastern, Mediterranean, Indian, and South Asian women with PCOS have a higher prevalence and/or severity of hirsutism than the East Asian or Caucasian (Finnish, Norwegian, and United States [US] White) women (6–10). These findings may be related to group level differences in genetic inheritance or expression patterns of the enzyme 5-*α* reductase, which converts testosterone to dihydrotestosterone, the more potent androgen implicated in PCOS pathogenesis (6, 8, 11). East Asian women have a low 5-*α* reductase activity likely contributing to their reduced severity of hirsutism (8). However, the severity of clinical hirsutism poorly correlates with the levels of circulating androgens, and thus ethnic-specific definitions of hirsutism have been suggested (8, 9, 12, 13).

Additionally, there are global differences in the prevalence of adverse metabolic outcomes associated with PCOS in women of different racial and ethnic groups (6, 7, 10). In a recent systematic review, 30 studies evaluated and compared metabolic outcomes of women with PCOS of different ethnicities worldwide (14). South Asian, Indian, and Norwegian women with PCOS in particular are at increased risk of developing metabolic syndrome (MetSyn), whereas Hispanic and Mexican women are at high risk of developing insulin resistance, and US Black women are at increased risk of hypertension compared with White women (7, 10, 14–16). In a cross-sectional study of over 1000 women with PCOS in 5 countries, Chan et al. (10) found a significant difference in the prevalence of MetSyn, as well as the clustering of its components in different racial/ethnic groups (10). Our current study aimed to determine the differences in phenotype and health disparities (defined by the Centers for Disease Control as preventable differences in the burden of disease, injury, violence, or in opportunities to achieve optimal health experienced by populations that have been disadvantaged by their social or economic status, geographic location, and environment) between Black, Hispanic, and Asian women compared with non-Hispanic White women with PCOS in the US (17).

## MATERIALS AND METHODS

**FIGURE 1:**
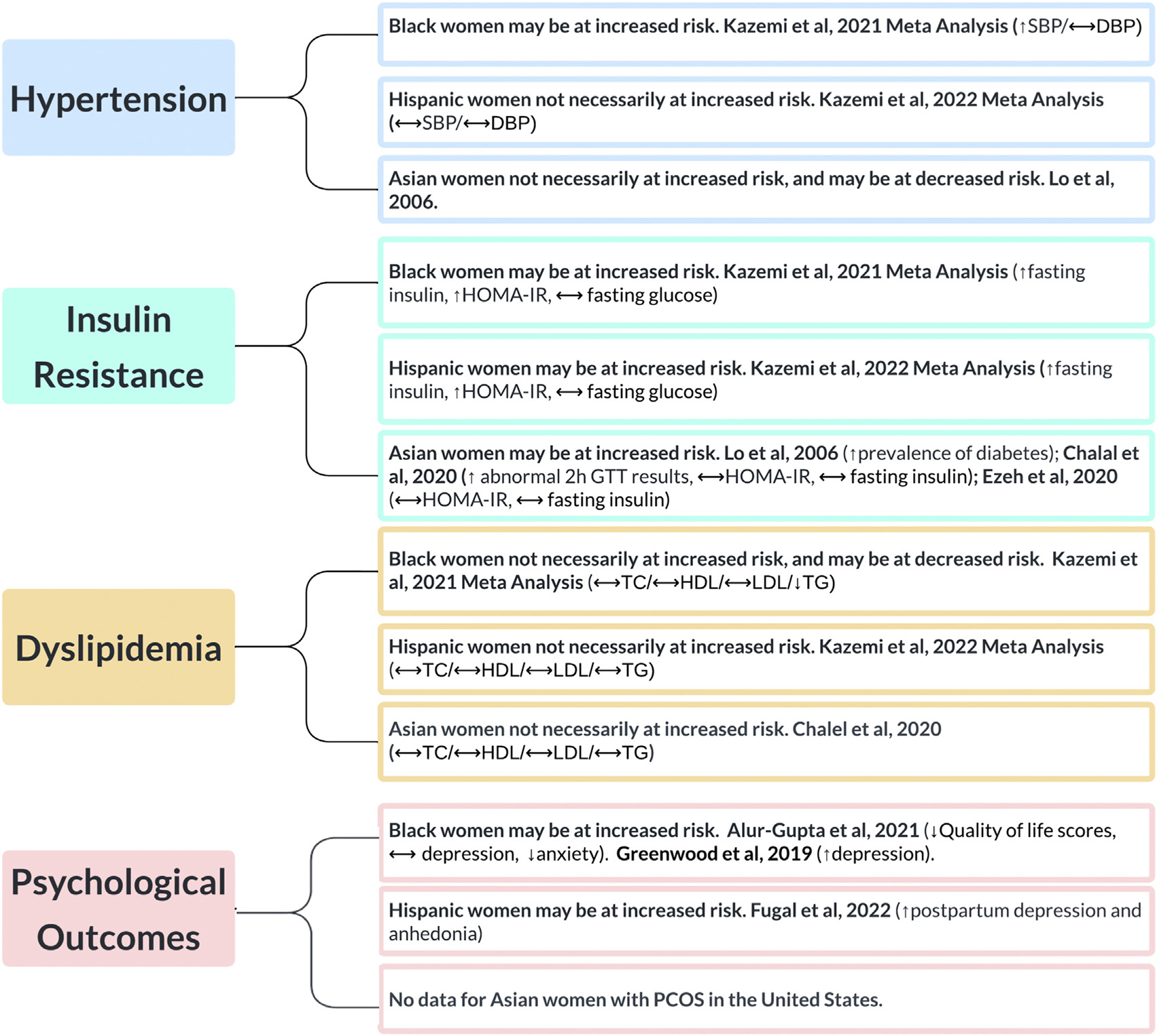
A summary of racial and ethnic disparities in women with PCOS in the United States. HOMA-IR = homeostatic model assessment of insulin resistance; SBP = systolic blood pressure; DBP = diastolic blood pressure; GTT = glucose tolerance test; TC = total cholesterol; HDL = high-density lipoprotein; LDL = low-density lipoprotein; TG = triglycerides; PCOS = polycystic ovary syndrome. VanHise. Racial and ethnic disparities in PCOS. Fertil Steril 2023.

All articles included in this review were obtained from a thorough search of the electronic databases PubMed, Web of Science, and Ovid MEDLINE. Search terms included “polycystic ovary syndrome or PCOS” and all of the following: “race, racial, ethnicity, ethnic, Black, Hispanic, Asian, White.” Articles were included if they were published in the English language, peer-reviewed, included the target population (women with PCOS in the US), addressed the objective (racial/ethnic disparities) and were published after the year 2000. We did not exclude articles based on the diagnostic criteria used for establishing a diagnosis of PCOS. For articles pertaining to disparities of Black and Hispanic women with PCOS, we chose to only include articles beyond the most recent systematic reviews and meta-analyses, which were published in 2021 and 2022, respectively (18, 19). The following data were extracted from primary articles: name of the first author, year of publication, study design, definition of PCOS, number of participants in the study, the average/median age of participants, the average/median body mass index (BMI) of participants, the outcomes measured, and results. A global summary of our findings is depicted in Figure 1.

## SUMMARY OF CURRENT EVIDENCE BY RACE/ETHNICITY

### Disparities in Risk for Black Women with PCOS in the US

A 2021 systematic review and meta-analysis of 11 studies evaluated the cardiometabolic risk profiles in Black (n = 652) and White (n = 2199) women with PCOS in the US (10, 19–29). Black women had increased fasting insulin levels, homeostatic model assessment of insulin resistance (HOMA-IR) scores, and systolic blood pressure when compared with White women (19). The heterogeneity of these findings was high, and could not be explained with meta-regression analyses for age, PCOS criteria, or ethnicity (19). A meta-regression analysis found that BMI was associated with systolic blood pressure (19). Black and White women with PCOS had comparable total cholesterol, low-density lipoprotein cholesterol, high-density lipoprotein cholesterol, fasting glucose, and diastolic blood pressures (19). There was a high degree of heterogeneity in these findings, with the exception of low-density lipoprotein cholesterol which had homogenous results (19). Black women had lower triglycerides compared with White women; however, this finding had a high degree of heterogeneity between studies which correlated with BMI (19). Overall these results suggest that Black women with PCOS have an increased risk of metabolic dysfunction associated with PCOS, although there is a high degree of heterogeneity between studies that is not always correlated with age or BMI.

A recent longitudinal cohort study of hyperandrogenic Black and White women with PCOS had similar metabolic findings to those outlined above: Black women with PCOS were more likely to have elevated blood pressure (45.1% vs. 33.8%, *P*=.10), and less likely to have elevated triglyceride levels when compared with White women with PCOS (5.4% vs. 25.3%, *P*<.01) (30). In this study women were observed for >3 years, during which time the rate of development of MetSyn (28% vs. 12%, *P*<.01) and overall incidence of MetSyn (45.9 ± 4.74 vs. 31.3 ± 3.03 per 100 person years) was higher in Black women with PCOS than in White women with PCOS, that persisted after adjustment for age and medication status (30). However, Black women had a significantly higher BMI than White women (average difference of 5.7 ± 1.2 kg/m^2^ throughout the study period), potentially confounding these results (30). The incidence of MetSyn significantly correlated with BMI in both races, suggesting that obesity plays a significant role in these findings.

In addition to the disparities in cardiometabolic outcomes, Black women with PCOS are also at increased risk of psychological comorbidities. In a recent cross-sectional study comparing racial differences in mental health metrics in patients with PCOS, Black women had lower modified PCOS quality-of-life survey scores in the infertility domain than those of White women, which remained significant after adjusting for age, BMI, and socioeconomic status (31). The prevalence of depression between Black and White women with PCOS in this study showed no significant differences, and White women with PCOS were more likely to have anxiety compared with Black women with PCOS (31). Somewhat contrary to these findings, results of another recent longitudinal cohort study observing women for 30 years found that Black women with PCOS had a higher depression burden than that of White women with PCOS, after accounting for age, BMI, race, education, and exercise output (32). Ongoing research is needed to address mental health disparities in Black women with PCOS.

### Disparities in Risk for Hispanic Women with PCOS in the US

The US Census Bureau considers Hispanic or Latino origin as an ethnicity and distinct concept from race (33). Individuals of Cuban, Mexican, Puerto Rican, South or Central American, or other Spanish culture or origin can be defined as Hispanic or Latino regardless of their race (33).

This distinction of race and ethnicity (e.g., differentiating Hispanic White vs. Hispanic Black) is not universally used and creates major limitations in research. Of note, despite the US Census Bureau categorization of people, the Pan American Health Organization/World Health Organization holds the scientifically accurate view that there is a single human race and considers differences between individuals to be of a cultural and symbolic nature and uses ethnicity to characterize sociocultural groups (34). That being said, as social constructs race and ethnicity can provide important group level insights into disparities and in rare cases group-level differences in gene polymorphisms (35).

A 2022 systematic review and meta-analysis included 11 studies comparing cardiometabolic and reproductive risks between Hispanic and White women with PCOS (18, 20, 23, 25, 26, 28, 29, 36–40). Hispanic women were more likely to have an elevated fasting insulin level and higher HOMA-IR scores than that of non-Hispanic White women in this study, with moderate heterogeneity which was not explained by age, BMI, or PCOS criteria (18). Hispanic and White women had comparable glucose, lipid profiles, and blood pressure (18). These results suggest an impaired glucoregulatory response in Hispanic women with PCOS, which may put these women at increased risk of metabolic sequelae such as type 2 diabetes. A recent retrospective chart review of female adolescents with overweight and obesity aged 11–21 years with PCOS found that Hispanic adolescents who had an elevated hemoglobin A_1c_ (HbA_1c_) and elevated alanine aminotransferase (ALT) levels at the time of PCOS diagnosis were at particularly higher risk of developing type 2 diabetes than Hispanic adolescents who had neither or only one of the elevated HbA_1c_ or ALT levels (hazard ratio, 19.0; 95% confidence interval [CI], 3.7–97.2; *P*<.001) (41). Current International PCOS Guidelines (1) do not recommend routine screening of ALT in PCOS, and thus additional research is needed to determine whether Hispanic adolescents with PCOS may benefit from targeted HbA_1c_ and ALT screening.

Hispanic women with PCOS are also at an increased risk of mental health disparities. A 2022 population-based study of postpartum women found that Hispanic women with a self-reported, prepregnancy diagnosis of PCOS or PCOS symptoms were at increased risk of postpartum depressed mood and anhedonia compared with their non-Hispanic counterparts (42).

### Disparities in Risk for Asian Women with PCOS in the US

Arguably one of the biggest limitations with research involving Asian individuals is that the term “Asian” is used to describe a very large and diverse group. According to the US Census Bureau, an individual of the Asian race includes any person “having origins in the original people of the Far East, Southeast Asia, or Indian subcontinent,” including (but not limited to). Cambodia, China, India, Japan, Korea, Malaysia, Pakistan, the Philippines, Thailand, and Vietnam (43, 44). There is substantial variation in the prevalence and phenotypic presentation of PCOS in women of different Asian ethnicities, so much so that ethnic-specific modified Ferriman-Gallwey cutoff scores have been proposed to define hirsutism in certain Asian populations (9). There is also evidence to suggest varying degrees of metabolic dysfunction (obesity, insulin resistance, and diabetes mellitus) among women with PCOS of different Asian and Asian American groups (8, 45, 46). There are only a limited number of studies in the PCOS literature pertaining to metabolic outcomes and associated risks for Asian women with PCOS in the US (26, 47–49). Results from a 2006 study in which women with PCOS were screened for eligibility for a clinical trial found that the prevalence of MetSyn did not differ significantly among racial and ethnic groups (Caucasian, African American, Hispanic, Asian, and mixed ancestral origin) (47). A 2006 retrospective cohort study performed in a large community-based health system in Northern California, including >7000 women with PCOS, found that Asian women with PCOS (n = 1117) were much less likely to be obese than other racial groups, but more likely to have diabetes than White women after adjustment for age and BMI (adjusted odds ratio [aOR], 2.16; 1.63–2.85) (48).

A recent cross-sectional study compared the metabolic outcomes in women of a single region of the US who self-identified as Asian, and further self-categorized into South Asian (n = 25) or East Asian (n = 38) based on their country of ethnic origin (49). East Asian women aged ≥25–30 years had elevated 2-hour insulin levels during an oral glucose tolerance test, whereas South Asian women aged ≥30 years had elevated 2-hour insulin levels when compared with White women (*P*=.03) (49). Additionally, 2-hour glucose levels were higher in East Asian women than in White women (*P*=.05) (49). All other metabolic outcomes (HOMA-IR, lipid panels, and fasting glucose) did not differ among racial and ethnic groups, and furthermore there was no increased risk of MetSyn between race groups in age-adjusted pairwise comparisons (49). The investigators concluded that additional studies with larger groups of East and South Asians would help elucidate whether these findings are clinically meaningful.

Another recent cross-sectional study compared markers of insulin resistance in women with PCOS of different self-reported race and ethnicities, including Asian Americans (n = 21) (26). Asian American women with PCOS had lower HOMA-IR scores and lower fasting and 2-hour an oral glucose tolerance test insulin levels than that of African American or Hispanic White women, including after adjustment for age and BMI (26). There was no statistically significant difference in the mean HOMA-IR scores between Asian American and non-Hispanic White women (*P*=.268). Asian American PCOS women had higher mean glucose values (although still normal) during a 2-hour OGT than that of non-Hispanic White women (60 min glucose 7.27 ± 0.49 vs. 6.46 ± 0.17, adjusted *P* value=.017; 120 min glucose 6.12 ± 0.28 vs. 5.40 ± 0.12, adjusted *P*=.008) (26). These results suggest that Asian American women may have a blunted insulin response at baseline or after an oral glucose challenge compared with non-Hispanic White women, but ongoing research including larger studies of Asian patients is necessary (26). Finally, there are no studies addressing mental health outcomes of Asian women with PCOS. This represents a major gap in knowledge and area for additional research.

## THE INTERSECTION OF GENETICS, THE ENVIRONMENT, CULTURE AND RACE/ETHNICITY

Our discussion on racial and ethnic differences in PCOS is incomplete without recognizing the potential impact of epigenetic, socioeconomic, cultural, or environmental factors on these results. For example, to what extent is a racial or ethnic group predisposed to developing an adverse PCOS outcome attributable to their continental or population ancestry (i.e., population level variations in PCOS related gene polymorphisms), for which race or ethnicity is often used as a poor surrogate, vs. their surrounding environment, including downstream psycho- neurohormonal, epigenetic, and behavioral responses to disparate treatment by society?

The potential impact of socioeconomic status on the risk of developing PCOS was studied in a cohort of >1000 White and Black women (50). Results of this study found that a low parental education level (considered ≤high school) was associated with an increased risk of PCOS (*P*=.03) (50). Interestingly, the highest risk group comprised women who achieved a high personal education level, but whose parents had a low education level; these findings were significant after adjustment for age, BMI, and race (aOR, 2.5; 95% CI, 1.4–4.4) (50). One limitation to this study is that other contributing factors to socioeconomic status (such as wealth, income, and occupation) were not assessed. There are racial and ethnic differences in many socioeconomic factors, including income, primary household occupation, household size, household location, and household expenditures in the US (51).

One example of differing household expenditures by race or ethnicity includes food purchasing habits. During the years 2014–2016, the average annual spending on fresh fruits in Black, Hispanic/Latino, American Indian/Alaskan Native, multiracial, Native Hawaiian/Pacific Islander, White, and Asian US households was $146.19, $319.06, $159.38, $232.79, $396.21, $238.51, and $408.16, respectively (51). This difference in spending is likely impacted by the cost of fruit in relation to other food products, family income, access to food retailers, the types of food available at food retailers, and cultural norms. The purchase and consumption of specific foods, such as fresh fruit, can impact many diseases, including PCOS. In the PCOS literature, starch and dairy products have been found to have negative metabolic impacts, whereas foods rich in inositiol compounds (such as fruit and beans) have beneficial effects on PCOS outcomes (52–54). Additionally, food purchasing decisions and diet are impacted by an individual’s geographic location within the US.

A 2019 study found that Healthy Eating Index scores varied across locations of the US, with the highest/healthiest scores reported in the West and the lowest/least healthy scores reported in the South (*P*<.05) (55). The specific food decisions that contributed to these findings varied by race (55). The intersection of the impact of environment and an individual’s race or ethnicity on PCOS is an important area of future study.

Merkin et al. (52) in their 2016 review, and the 2012 Amsterdam European Society of Human Reproduction and Embryology/American Society of Reproductive Medicine PCOS Workshop outlined the need for ongoing investigation into the “role of genetic and environmental factors to explain ethnic variances” of PCOS, and suggested a strategy to “study populations of similar races and ethnicities across geographic regions with the use of the same tools and protocols (7, 52).” We aimed to address the objectives set forth by Merkin et al. (52) and the European Society of Human Reproduction and Embryology/American Society of Reproductive Medicine PCOS Workshop in our recent study comparing Black and White women with PCOS in 2 unique geographic areas of the US (Birmingham, Alabama and Los Angeles, California). We found that Alabama women of both races were at an increased risk of insulin resistance compared with California women, even after adjustment for age and BMI (56). The only significant difference between Black and White women in our study was that Black patients with PCOS had no statistically significant difference in average BMI between the 2 locations; in the White cohort, Alabama women had a higher BMI (56). We hypothesized that differences in normative diets, exposure to environmental toxins, socioeconomic status, and family ancestry of individuals of the same race residing in the 2 locations contributed to these results (56). Another important consideration is that the participants included in the aforementioned studies were recruited and/or referred to appropriate health care providers. This creates an inherent bias in the study populations, which is reflective of inequities of our health care system, and social/cultural norms of seeking out care for symptoms of PCOS (i.e., androgen excess or infertility). One example highlighting referral bias of our health care system is a 2013 study that compared features of women who were referred for evaluation of PCOS with the features of women who were identified to have PCOS on a preemployment physical examination (i.e., an unselected population). The investigators found that 84% of women in the referral group were non-Hispanic White women, and in comparison, 53% of women identified in the nonreferral groups were African American women (57). There were similar findings in a 2011 study of US women seeking infertility care; African American, Asian American, and Hispanic women were more likely to be self-referred or referred by a friend or family member when compared with White women, who were more likely to be referred by a physician (58). Results of this study also suggested racial and ethnic differences in concerns for seeking out infertility care; for example, African American, Asian American, and in particular, Chinese American women, were more concerned about the social stigma of infertility than White women (58). These results highlight the idea of a “culture of silence,” in which some women may not seek care for diseases such as infertility and PCOS.

A potential consequence of disparate health seeking behaviors and access to health care is an incomplete cohort of women with PCOS in research, thus limiting the ability to form more accurate conclusions on racial and ethnic associated PCOS phenotypes and metabolic outcomes. This is also reflected by the substantial difference in the number of White/Non-Hispanic subjects vs. number of non-White and/or Hispanic subjects included in studies. Despite the similar prevalence of PCOS between White (4.7%) and Black (3.5%) women in an unselected population of women (59), Black women are numerically underrepresented in PCOS literature.

Similarly, Asian women are underrepresented in PCOS literature, with many studies not including the Asian race at all, or having such a small number of Asian women that they are not included in comparative analyses. Additionally, there are extremely limited or nonexistent PCOS data on racial and ethnic groups, such as American Indians, Pacific Islanders, Native Hawaiians, Alaska Natives, and multiracial individuals. Additionally, more studies comparing different world regions outside of the US would also be critical in understanding the role that race and ethnicity play on expression of the syndrome. Future work is needed to fill these voids in our understanding of PCOS.

## References

[1] Teede HJ, Misso ML, Costello MF, Dokras A, Laven J, Moran L, Recommendations from the international evidence-based guideline for the assessment and management of polycystic ovary syndrome. Fertil Steril 2018;110:364–79. 30033227 10.1016/j.fertnstert.2018.05.004 PMC6939856

[2] Vink JM, Sadrzadeh S, Lambalk CB, Boomsma DI. Heritability of polycystic ovary syndrome in a Dutch twin-family study. J Clin Endocrinol Metab 2006;91:2100–4. 16219714 10.1210/jc.2005-1494

[3] Hiam D, Moreno-Asso A, Teede HJ, Laven JSE, Stepto NK, Moran LJ, The genetics of polycystic ovary syndrome: an overview of candidate gene systematic reviews and genome- wide association studies. J Clin Med 2019;8. 10.3390/jcm8101606 PMC6832583 31623391

[4] Chen ZJ, Zhao H, He L, Shi Y, Qin Y, Shi Y, Genome-wide association study identifies susceptibility loci for polycystic ovary syndrome on chromosome 2p16.3, 2p21 and 9q33.3. Nat Genet 2011;43:55–9. 21151128 10.1038/ng.732

[5] Goodarzi MO, Jones MR, Li X, Chua AK, Garcia OA, Chen YD, Replication of association of DENND1A and THADA variants with polycystic ovary syndrome in European cohorts. J Med Genet 2012;49:90–5. 22180642 10.1136/jmedgenet-2011-100427 PMC3536488

[6] Glintborg D, Mumm H, Hougaard D, Ravn P, Andersen M. Ethnic differences in Rotterdam criteria and metabolic risk factors in a multiethnic group of women with PCOS studied in Denmark. Clin Endocrinol (Oxf) 2010;73:732–8. 20846294 10.1111/j.1365-2265.2010.03873.x

[7] Fauser BC, Tarlatzis BC, Rebar RW, Legro RS, Balen AH, Lobo R, Consensus on women’s health aspects of polycystic ovary syndrome (PCOS): the Amsterdam ESHRE/ASRM-Sponsored 3rd PCOS Consensus Workshop Group. Fertil Steril 2012;97:28–38.e25. 22153789 10.1016/j.fertnstert.2011.09.024

[8] Zhao Y, Qiao J. Ethnic differences in the phenotypic expression of polycystic ovary syndrome. Steroids 2013;78:755–60. 23624030 10.1016/j.steroids.2013.04.006

[9] Huang Z, Yong EL. Ethnic differences: is there an Asian phenotype for polycystic ovarian syndrome? Best Pract Res Clin Obstet Gynaecol 2016;37:46–55. 27289337 10.1016/j.bpobgyn.2016.04.001

[10] Chan JL, Kar S, Vanky E, Morin-Papunen L, Piltonen T, Puurunen J, Racial and ethnic differences in the prevalence of metabolic syndrome and its components of metabolic syndrome in women with polycystic ovary syndrome: a regional cross-sectional study. Am J Obstet Gynecol 2017;217:189.e1–.e8. 10.1016/j.ajog.2017.04.007 28400308

[11] Goodarzi MO, Shah NA, Antoine HJ, Pall M, Guo X, Azziz R. Variants in the 5alpha-reductase type 1 and type 2 genes are associated with polycystic ovary syndrome and the severity of hirsutism in affected women. J Clin Endocrinol Metab 2006;91:4085–91. 16849416 10.1210/jc.2006-0227

[12] Azziz R, Carmina E, Sawaya ME. Idiopathic hirsutism. Endocr Rev 2000;21:347–62. 10950156 10.1210/edrv.21.4.0401

[13] Kiconco S, Mousa A, Azziz R, Enticott J, Suturina LV, Zhao X, PCOS Phenotype in Unselected Populations Study (P-PUP): protocol for a systematic review and defining pcos diagnostic features with pooled individual participant data. Diagnostics (Basel) 2021:11. 10.3390/diagnostics11111953 PMC8618006 34829300

[14] Sendur SN, Yildiz BO. Influence of ethnicity on different aspects of polycystic ovary syndrome: a systematic review. Reprod Biomed Online 2021;42:799–818. 33487557 10.1016/j.rbmo.2020.12.006

[15] Wijeyaratne CN, Balen AH, Barth JH, Belchetz PE. Clinical manifestations and insulin resistance (IR) in polycystic ovary syndrome (PCOS) among South Asians and Caucasians: is there a difference? Clin Endocrinol (Oxf) 2002;57:343–50. 12201826 10.1046/j.1365-2265.2002.01603.x

[16] Kauffman RP, Baker VM, Dimarino P, Gimpel T, Castracane VD. Polycystic ovarian syndrome and insulin resistance in White and Mexican American women: a comparison of two distinct populations. Am J Obstet Gynecol 2002;187:1362–9. 12439532 10.1067/mob.2002.126650

[17] Centers for Disease Control and Prevention. What is health equity? [updated July 1, 2022. Available from: https://www.cdc.gov/healthequity/whatis/index.html. Accessed Dec 1, 2022.

[18] Kazemi M, Kim JY, Wan C, Xiong JD, Parry SA, Azziz R, Comprehensive evaluation of disparities in cardiometabolic and reproductive risk between Hispanic and White women with polycystic ovary syndrome in the United States: a systematic review and meta-analysis. Am J Obstet Gynecol 2022;226:187–204.e15. 34384776 10.1016/j.ajog.2021.07.032

[19] Kazemi M, Kim JY, Parry SA, Azziz R, Lujan ME. Disparities in cardio metabolic risk between Black and White women with polycystic ovary syndrome: a systematic review and meta-analysis. Am J Obstet Gynecol 2021;224:428–44.e8. 33316275 10.1016/j.ajog.2020.12.019

[20] Chang AY, Oshiro J, Ayers C, Auchus RJ. Influence of race/ethnicity on cardiovascular risk factors in polycystic ovary syndrome, the Dallas Heart Study. Clin Endocrinol (Oxf) 2016;85:92–9. 26608823 10.1111/cen.12986 PMC4882287

[21] Hillman JK, Johnson LN, Limaye M, Feldman RA, Sammel M, Dokras A. Black women with polycystic ovary syndrome (PCOS) have increased risk for metabolic syndrome and cardiovascular disease compared with White women with PCOS [corrected]. Fertil Steril 2014;101:530–5. 24382375 10.1016/j.fertnstert.2013.10.055

[22] Ladson G, Dodson WC, Sweet SD, Archibong AE, Kunselman AR, Demers LM, Racial influence on the polycystic ovary syndrome phenotype: a black and white case-control study. Fertil Steril 2011;96:224–9.e2. 21723443 10.1016/j.fertnstert.2011.05.002 PMC3132396

[23] Welt CK, Arason G, Gudmundsson JA, Adams J, Palsdóttir H, Gudlaugsdóttir G, Defining constant versus variable phenotypic features of women with polycystic ovary syndrome using different ethnic groups and populations. J Clin Endocrinol Metab 2006;91:4361–8. 16940441 10.1210/jc.2006-1191

[24] Ehrmann DA, Kasza K, Azziz R, Legro RS, Ghazzi MN. Effects of race and family history of type 2 diabetes on metabolic status of women with polycystic ovary syndrome. J Clin Endocrinol Metab 2005;90:66–71. 15507516 10.1210/jc.2004-0229

[25] Engmann L, Jin S, Sun F, Legro RS, Polotsky AJ, Hansen KR, Racial and ethnic differences in the polycystic ovary syndrome metabolic phenotype. Am J Obstet Gynecol 2017;216:493.e1–.e13. 10.1016/j.ajog.2017.01.003 PMC5420474 28104402

[26] Ezeh U, Ida Chen YD, Azziz R. Racial and ethnic differences in the metabolic response of polycystic ovary syndrome. Clin Endocrinol (Oxf) 2020;93:163–72. 32286715 10.1111/cen.14193

[27] Koval KW, Setji TL, Reyes E, Brown AJ. Higher high-density lipoprotein cholesterol in African-American women with polycystic ovary syndrome compared with Caucasian counterparts. J Clin Endocrinol Metab 2010;95:E49–53. 20534766 10.1210/jc.2010-0074 PMC2936063

[28] Legro RS, Myers ER, Barnhart HX, Carson SA, Diamond MP, Carr BR, The Pregnancy in polycystic ovary syndrome study: baseline characteristics of the randomized cohort including racial effects. Fertil Steril 2006;86:914–33. 16963034 10.1016/j.fertnstert.2006.03.037

[29] Sam S, Scoccia B, Yalamanchi S, Mazzone T. Metabolic dysfunction in obese Hispanic women with polycystic ovary syndrome. Hum Reprod 2015;30:1358–64. 25857311 10.1093/humrep/dev073 PMC4498223

[30] Lee I, Vresilovic J, Irfan M, Gallop R, Dokras A. Higher incidence of metabolic syndrome in Black women with polycystic ovary syndrome: a longitudinal study. J Clin Endocrinol Metab 2022;107:e1558–67. 34928388 10.1210/clinem/dgab840

[31] Alur-Gupta S, Lee I, Chemerinski A, Liu C, Lipson J, Allison K, Racial differences in anxiety, depression, and quality of life in women with polycystic ovary syndrome. F S Rep 2021;2:230–7. 34278359 10.1016/j.xfre.2021.03.003 PMC8267396

[32] Greenwood EA, Yaffe K, Wellons MF, Cedars MI, Huddleston HG. Depression over the lifespan in a population-based cohort of women with polycystic ovary syndrome: longitudinal analysis. J Clin Endocrinol Metab 2019;104:2809–19. 30985868 10.1210/jc.2019-00234 PMC6534493

[33] Hispanic or Latino Origin US Census Bureau. Population estimates program. Available From: https://www.census.gov/quickfacts/fact/note/US/RHI725221. Accessed Dec 1, 2022.

[34] Ethnicity and Health. Pan American Health Organization/World Health Organization.132nd Session of the Executive Committee. 2003. Available from: http://www1.paho.org/english/gov/ce/ce132-16-e.pdf. Accessed Dec 1, 2022.

[35] Mohottige D, Boulware LE, Ford CL, Jones C, Norris KC. Use of race in kidney research and medicine: concepts, principles, and practice. Clin J Am Soc Nephrol 2022;17:314–22. 34789476 10.2215/CJN.04890421 PMC8823929

[36] Dunaif A, Sorbara L, Delson R, Green G. Ethnicity and polycystic ovary syndrome are associated with independent and additive decreases in insulin action in Caribbean-Hispanic women. Diabetes 1993;42:1462–8. 8375585 10.2337/diab.42.10.1462

[37] Hara M, Alcoser SY, Qaadir A, Beiswenger KK, Cox NJ, Ehrmann DA. Insulin resistance is attenuated in women with polycystic ovary syndrome with the Pro(12)Ala polymorphism in the PPARgamma gene. J Clin Endocrinol Metab 2002;87:772–5. 11836319 10.1210/jcem.87.2.8255

[38] Kauffman RP, Baker VM, DiMarino P, Castracane VD. Hyperinsulinemia and circulating dehydroepiandrosterone sulfate in White and Mexican American women with polycystic ovary syndrome. Fertil Steril 2006;85:1010–6. 16580388 10.1016/j.fertnstert.2005.09.046

[39] Kauffman RP, Baker TE, Graves-Evenson K, Baker VM, Castracane VD. Lipoprotein profiles in Mexican American and non-Hispanic White women with polycystic ovary syndrome. Fertil Steril 2011;96:1503–7. 21982731 10.1016/j.fertnstert.2011.09.025

[40] Segal S, Elmadjian M, Takeshige T, Karp S, Mercado R, Rivnay B. Serum inhibin A concentration in women with polycystic ovarian syndrome and the correlation to ethnicity, androgens and insulin resistance. Reprod Biomed Online 2010;20:675–80. 20231113 10.1016/j.rbmo.2010.02.006

[41] Hudnut-Beumler J, Kaar JL, Taylor A, Kelsey MM, Nadeau KJ, Zeitler P, Development of type 2 diabetes in adolescent girls with polycystic ovary syndrome and obesity. Pediatr Diabetes 2021;22:699–706. 33870630 10.1111/pedi.13206 PMC8808365

[42] Fugal AD, Stanford JB, Johnstone EB, Kah K, Schliep KC. Polycystic ovary syndrome and postpartum depression among Hispanics and non-Hispanics: a population-based study. AJOG Glob Rep 2022;2:100070. 36060826 10.1016/j.xagr.2022.100070 PMC9438401

[43] Vu MH, Nguyen AA, Alur-Gupta S. Asian Americans and infertility: genetic susceptibilities, sociocultural stigma, and access to care. F S Rep 2022;3(Suppl 2):40–5. 35937455 10.1016/j.xfre.2021.12.004 PMC9349240

[44] United States Census Bureau. About the topic of race [updated March 1, 2022] Available from: https://www.census.gov/topics/population/race/about.html. Accessed Dec 1, 2022.

[45] Guo M, Chen ZJ, Eijkemans MJ, Goverde AJ, Fauser BC, Macklon NS. Comparison of the phenotype of Chinese versus Dutch Caucasian women presenting with polycystic ovary syndrome and oligo/amenorrhoea. Hum Reprod 2012;27:1481–8. 22402209 10.1093/humrep/des018

[46] Wijeyaratne CN, Seneviratne Rde A, Dahanayake S, Kumarapeli V, Palipane E, Kuruppu N, Phenotype and metabolic profile of South Asian women with polycystic ovary syndrome (PCOS): results of a large database from a specialist Endocrine Clinic. Hum Reprod 2011;26:202–13. 21098627 10.1093/humrep/deq310

[47] Ehrmann DA, Liljenquist DR, Kasza K, Azziz R, Legro RS, Ghazzi MN. Prevalence and predictors of the metabolic syndrome in women with polycystic ovary syndrome. J Clin Endocrinol Metab 2006;91:48–53. 16249284 10.1210/jc.2005-1329

[48] Lo JC, Feigenbaum SL, Yang J, Pressman AR, Selby JV, Go AS. Epidemiology and adverse cardiovascular risk profile of diagnosed polycystic ovary syndrome. J Clin Endocrinol Metab 2006;91:1357–63. 16434451 10.1210/jc.2005-2430

[49] Chahal N, Quinn M, Jaswa EA, Kao CN, Cedars MI, Huddleston HG. Comparison of metabolic syndrome elements in White and Asian women with polycystic ovary syndrome: results of a regional, American cross-sectional study. F S Rep 2020;1:305–13. 34223261 10.1016/j.xfre.2020.09.008 PMC8244318

[50] Merkin SS, Azziz R, Seeman T, Calderon-Margalit R, Daviglus M, Kiefe C, Socioeconomic status and polycystic ovary syndrome. J Womens Health (Larchmt) 2011;20:413–9. 21323584 10.1089/jwh.2010.2303 PMC3115419

[51] Noël RA. Race, economics and social status. US Bureau of Labor Statistics 2018. Available from: https://www.bls.gov/spotlight/2018/race-economics-and-social-status/pdf/race-economics-and-social-status.pdf.

[52] Merkin SS, Phy JL, Sites CK, Yang D. Environmental determinants of polycystic ovary syndrome. Fertil Steril 2016;106:16–24. 27240194 10.1016/j.fertnstert.2016.05.011

[53] Plotan M, Elliott CT, Frizzell C, Connolly L. Estrogenic endocrine disruptors present in sports supplements. A risk assessment for human health. Food Chem 2014;159:157–65. 24767039 10.1016/j.foodchem.2014.02.153

[54] Phy JL, Pohlmeier AM, Cooper JA, Watkins P, Spallholz J, Harris KS, Low starch/low dairy diet results in successful treatment of obesity and comorbidities linked to polycystic ovary syndrome (PCOS). J Obes Weight Loss Ther 2015;5:259. 26225266 10.4172/2165-7904.1000259 PMC4516387

[55] Vadiveloo M, Perraud E, Parker HW, Juul F, Parekh N. Geographic differences in the dietary quality of food purchases among participants in the nationally representative Food Acquisition and Purchase Survey (FoodAPS). Nutrients 2019;11:1233. 31151225 10.3390/nu11061233 PMC6627193

[56] VanHise K, Chan JL, Wertheimer S, Handelsman RG, Clark E, Buttle R, Regional variation in hormonal and metabolic parameters of White and Black women with PCOS in the United States. J Clin Endocrinol Metab 2022;:dgac515. 10.1210/clinem/dgac515 PMC10210617 36218376

[57] Ezeh U, Yildiz BO, Azziz R. Referral bias in defining the phenotype and prevalence of obesity in polycystic ovary syndrome. J Clin Endocrinol Metab 2013;98:E1088–96. 23539721 10.1210/jc.2013-1295 PMC3667270

[58] Missmer SA, Seifer DB, Jain T. Cultural factors contributing to health care disparities among patients with infertility in Midwestern United States. Fertil Steril 2011;95:1943–9. 21420677 10.1016/j.fertnstert.2011.02.039

[59] Knochenhauer ES, Key TJ, Kahsar-Miller M, Waggoner W, Boots LR, Azziz R. Prevalence of the polycystic ovary syndrome in unselected black and white women of the southeastern United States: a prospective study. J Clin Endocrinol Metab 1998;83:3078–82. 9745406 10.1210/jcem.83.9.5090

